# *Lactobacillus plantarum* 5BG Survives During Refrigerated Storage Bio-Preserving Packaged Spanish-Style Table Olives (cv. Bella di Cerignola)

**DOI:** 10.3389/fmicb.2018.00889

**Published:** 2018-05-07

**Authors:** Paola Lavermicocca, Luisa Angiolillo, Stella L. Lonigro, Francesca Valerio, Antonio Bevilacqua, Marianne Perricone, Matteo A. Del Nobile, Maria R. Corbo, Amalia Conte

**Affiliations:** ^1^Institute of Sciences of Food Production, National Research Council, Bari, Italy; ^2^Department of the Science of Agriculture, Food and Environment, University of Foggia, Foggia, Italy

**Keywords:** olives, Bella di Cerignola, bio-preservation, quality, safety

## Abstract

This paper proposes bio-preservation as a tool to assure quality and safety of Spanish-style table olives cv. Bella di Cerignola. *Lactobacillus plantarum* 5BG was inoculated in ready to sell olives packaged in an industrial plant by using a half-volume brine (4% NaCl; 2% sucrose). The samples were stored at 4°C. The survival of the inoculated strain, the microbiological quality, the sensory scores and the survival of a strain of *Listeria monocytogenes* inoculated in brines were evaluated. The persistence of the *Lb. plantarum* bio-preserving culture was confirmed on olives (≥6.5 Log CFU/g) and in brine (≥7 Log CFU/ml). Bio-preserved olives (SET1) showed a better sensory profile than chemically acidified control olives (SET2) and the texture was the real discriminative parameter among samples. Bio-preserved olives recorded better scores during storage because of their ability to retain good hardness, crunchiness, and fibrousness without cracks. The inoculation of *Lb. plantarum* positively acted on the safety of olives, as the *D*-value of *L. monocytogenes* was reduced from 40 (SET2) to 5 days (SET1). In conclusion, *Lb. plantarum* 5BG and the physico-chemical conditions achieved in the settled procedure are suitable for the industrial packaging of Bella di Cerignola table olives, improving the process by halving brining volumes and avoiding chemical stabilizers, and significantly reducing the salt concentration. The final product is also safely preserved for almost 5 months as suggested by the reduction of the survival rate of *L. monocytogenes*.

## Introduction

Bio-preservation, defined as the extension of shelf life and food safety by the use of microbial cultures, has been used to ensure high sensory and functional quality of several products i.e., dairy, meat, and vegetables including fermented olives (Ananou et al., 2007; Liu et al., 2008; Trias et al., 2008; De Bellis et al., 2010).

According to the most well-known “Spanish-style” processing method, Spanish-style table olives cv. Bella di Cerignola are debittered and brined in 12–13% NaCl where they undergo spontaneous fermentation by a mixed microbiota mainly composed by lactic acid bacteria (LAB) and yeasts. At the end of the process, fermentation is finished and pH decreases to <4.0 ensuring the preservation of the product. Olives may be stored in bulk in brine with NaCl∼10% and pH <4.0 or, after washing of fruits with water, packaged in small glass containers covered with fresh acidified brine and pasteurized to avoid undesirable fermentation that may affect the quality and safety of the product (Garrido-Fernández et al., 1997). Recently, some packaging solutions as plastic containers or tins are increasingly adopted instead of glass jars to prevent product alteration and to facilitate product managing and logistic organization (Sánchez et al., 2017). In the case of packaging in plastic pouches, the product is stabilized by adding chemical acidifiers and antioxidants with an expiry date no longer than 1 year, while refrigerated storage is recommended after package opening if product is not consumed in a single seating.

Table olives should maintain specific sensory standards as established by the Codex Alimentarius (CODEX/COI, 2013). Particularly, high flesh to stone ratio and a uniform size are the most apparent sensorial attributes. In addition, a firm texture and a color characteristic of the variety (that is green/strawy green for green olives) without white spots or color browning with an equilibrate taste are essential and deeply related to the microbial quality.

To meet consumers and producers demand for alternatives to chemical additives, the ability of a selected *Lactobacillus plantarum* in bio-preserving Spanish-style olives cv. Bella di Cerignola was evaluated in an industrial plant in retail packages. Bioprotective LAB cultures and/or their antimicrobial compounds have already been used to extend shelf life and ensure food safety (Leverentz et al., 2006). The strains used for bio-preservation belong to *Lactobacillus* species and they could generally exert a bacteriostatic or a bactericidal effect, because of the production of bacteriocins and other antimicrobial compounds and/or the competition for nutrients (Gálvez et al., 2010; Bevilacqua et al., 2015).

Particularly, Spanish-style olives fermented with *Lactobacillus pentosus* or *Lb. plantarum* cultures have been stored, using polyethylene (PE) pouches, in modified atmosphere (Argyri et al., 2015) or in brine (Blana et al., 2016). However, in these studies, LAB were applied as starter cultures at the onset of fermentation.

During storage, several pathogens could be found in olives and among them, *Listeria monocytogenes* has been frequently isolated (Caggia et al., 2004; Argyri et al., 2013; Panagou et al., 2013).

In particular, *L. monocytogenes* could survive in brines, as the combinations of pH, salt, and some additives used by producers could enhance its survival for months (Bevilacqua et al., 2018). The exposure of *L. monocytogenes* to a nonlethal pH (4.4÷5.8) for some hours, as may occur in fermented foods, can result in a higher pathogen survival that is of particular concern in products with an extended shelf life as the pathogen well adapts to the conditions used in manufacturing process (exposure to low temperatures, low pH, and elevated salt concentrations) developing defense mechanisms to tolerate these stresses (Farber and Pagotto, 1992; Gandhi and Chikindas, 2007; Uyttendaele et al., 2009; Bevilacqua et al., 2018). To face the risk of pathogen survival, the application of LAB populations as bio-preservatives contribute to warranty quality and safety of vegetable products (Valerio et al., 2013; Iglesias et al., 2017, 2018; Yépez et al., 2017).

The main goal of this research was to use a bio-preserving strain of *Lb. plantarum* during refrigerated storage in order to assure the microbiological and sensorial quality of Spanish-style olives cv. Bella di Cerignola packaged in plastic bags, containing low amount of brine with a minimum salt level and without chemical preservatives. In addition, the ability of the bio-preserving strain to counteract *Listeria monocytogenes* during storage was evaluated.

## Materials and Methods

### Olive Processing and Packaging

Spanish-style table olives used in the present study (cv. Bella di Cerignola), supplied by Cannone Industrie Alimentari SpA, were picked by hand at the green maturation stage during the 2015 crop season and prepared following the Spanish-style method. Olives were placed into tanks and covered with a 1.9% (w/v) NaOH solution for 8 h until the lye penetrated 2/3 of the pit; then fruits were washed with tap water for 10–12 h. Spontaneous fermentation, i.e., fermentation carried out by the indigenous microflora, was performed in plastic tanks keeping fruits (140 kg) submerged in brines (90 kg, 11% NaCl) by the means of perforated caps. After the fermentation, the tanks were stored for 1 year at room temperature increasing the salt concentration in brine up to 10%. *Lb. plantarum* 5BG (belonging to ISPA Collection n°ITEM 17403) was previously isolated from olive brines (Lavermicocca et al., 1998) and used in this study as freeze-dried powder. About 100 kg of olives were withdrawn from tanks, desalted (final NaCl 5%) by washing olives two times with tap water (about 100 L) and two experimental sets were settled up: (1) bio-preserved SET1 was obtained by adding the strain (Log 8 CFU/g of olives) in fresh brine (4% NaCl, 2% sucrose; pH 6.6) for 5 days at 25°C; (2) experimental SET2 was obtained by directly adding desalted olives to a chemically acidified brine (4% NaCl; 0.35% lactic acid; 0.35% citric acid, 0.35% ascorbic acid; pH <4.0).

Olives (400 g) from both sets were packaged in PE bags in the presence of 50 g of brine to obtain olive/brine ratio of 8/1 which was selected in preliminary experiments (unpublished results); bags were heat-sealed using an industrial-scale packaging machine (Tecnovac, Grassobbio, BG, Italy) and kept at 4°C for 1 year. Two independent experiments were performed and, for each analysis and sampling time, two separate bags for each set were prepared. In the case of microbiological and physico-chemical analyses, both brines and olives were analyzed.

### Physico-Chemical Analyses

The pH analyses of cover brines and olive fruits were carried out as described in De Bellis et al. (2010); results were the average of 4 and 12 measurements for brines and olives, respectively. The instrumental determination of firmness of olives was determined using a Kramer shear compression cell fixed to a texture analyzer Machine (Zwickiline Z0.5, Zwick/Roell, Ulm, Germany). Olives from each bag were manually pitted and cut longitudinally, resulting in one piece (about 10 mm × 10 mm × 5 mm) of flesh per olive. The olive pieces (about 1 gr each) were placed in the Kramer cell with the olive surface facing upward. The cross-head speed was set at 400 mm/min and the penetration force was measured in Newton (N). Data were collected and analyzed using the system software (Texture Expert). The maximum force required to penetrate olive section was recorded and firmness was calculated by dividing the maximum shear compression force with the total weight of olive pieces and expressed as N/g of product. Firmness values were the mean of 12 measurements, each of which from 12 different fruits.

The instrumental surface color analyses on the olive fruits was carried out using a Minolta CR-300 (Minolta Ltd., Osaka, Japan) colorimeter. The instrument was calibrated using a reference white tile and color was recorded in the CIE L^∗^ (lightness/brightness), a^∗^ (redness/greenness; negative values are related to green tones while positive values are associated to red tones) and b^∗^ (yellowness/blueness, representing colors on a blue (-) to yellow (+) axis) color scale. Total color difference (ΔE^∗^) was calculated as

$$\sqrt{\left[{\left(\Delta L*\right)}^{2}+{\left(\Delta a*\right)}^{2}+{\left(\Delta b*\right)}^{2}\right]}$$

using the color of olives before packaging as a reference

For each sample, 9 different olives from 2 different bags were analyzed to evaluate the skin color. Results were the average of 18 measurements.

### Microbiological Analyses

At each sampling time, two bags per set were opened and olive and brine samples were analyzed. Microorganisms adhering to the olive epidermis were determined as follows: at each sampling 50 g of olives each were pitted with a sterile knife under a laminar flow cabinet; 15 g portions from each replicate were homogenized in 135 g of sterile Buffered Peptone Water (BPW, Difco) for 1 min in a Blender (Waring). Decimal dilutions (100 μl or otherwise specified) were plated in duplicate on specific agar media. For LAB enumeration, dilutions were plated onto de Man–Rogosa–Sharpe medium (MRS) and incubated into a 9% CO_2_ incubator (Incusafe, Panasonic) at 37°C for 48 h; for total mesophilic count, dilutions were plated on plate count agar (PCA) and incubated at 30°C in aerobiosis; for yeasts and molds, dilutions were plated on Sabouraud Dextrose Agar (SDA) supplemented with chloramphenicol and chlortetracycline (both 0.05 g/l) and incubated at 25°C for 48 h and 5 days, respectively; total counts of Enterobacteriaceae were obtained by pour-plating dilutions (1 ml) in Violet Red Bile Glucose agar (VRBGA), incubated at 37°C for 24 h; Baird-Parker Agar, supplemented with egg yolk tellurite emulsion and incubated at 37°C for 48 h was used for *Staphylococcus* spp. Brines were diluted in a sterile saline solution (9 g/l NaCl) and plated on the same media.

### Model Development

To determine the microbial acceptability limit, a modified version of the Gompertz equation was fitted to the experimental data, as reported in previous works (Conte et al., 2009; Del Nobile et al., 2009):

$$\log(N\left(t\right))=\log(N_{\max})-A\cdot \exp\left[-\exp\left\{[\mu_{max}\cdot 2.71\frac{\lambda -MAL}{A}]+1\right\}\right]+A\cdot \exp[-\exp\left\{[\mu_{max}\cdot 2.71\frac{\lambda -t}{A}]+1\right\}]$$

Where *N*(*t*) is the viable cell concentration (CFU/g) at time *t*, *A* is related to the difference between the decimal logarithm of maximum bacterial growth attained at the stationary phase and the decimal logarithm of the initial cell load concentration (CFU/g), μ_max_ is the maximum growth rate [Log(CFU/g)/day], λ is the lag time (day), *t* is the time (day), *N*_max_ is the break-point (CFU/g), MAL is the microbial acceptability limit or microbiological shelf life (day) (i.e., the storage time at which the population attains the break point).

There are not specific actual microbiological standards for table olives. However, they can be sold in both bulk form in fermentation brine (unpasteurized) and in packages intended for retail sale (pasteurized or possibly unpasteurized); therefore, the limits for ready-to-eat products were used (10^6^ CFU/g for yeasts and 10^4^ CFU/g for Enterobacteriaceae and *Staphylococcus* spp.) (Food Safety Authority of Ireland, 2001; International Olive Council [IOC], 2004; Commission Regulation [EC], 2007).

The survival of the inoculated strain on olive drupes and in brines during storage was determined as follows: 20% of total presumptive LAB colonies (at least 10 colonies), randomly picked from countable MRS agar plates containing from 50 to 100 colonies, were isolated and checked for purity. Bacterial DNA from each colony was extracted from overnight cultures grown in MRS broth at 37°C as previously described (De Bellis et al., 2010). Genotypic identification of *Lb. plantarum* 5BG was based on the comparison of the REP-PCR profile of each isolate with the specific pattern obtained from the pure culture of *Lb. plantarum* 5BG strain. The amplification products were separated by the Lab-on-a-Chip (LoaC) capillary electrophoresis carried out on a 2100 Bioanalyzer from Agilent Technologies using the DNA 7500 LabChip kit (Agilent Technologies, Waldbronn, Germany). Sample preparation and chip loading was performed according to manufacturer’s instructions. DNA fragments were separated electrophoretically and data elaborated by 2100 Expert Software were automatically visualized as peaks in an electropherogram and bands in a gel-like image. Each chip included a sizing ladder containing 10 reference fragments ranging from 50 to 7,000 bp flanked by an upper (10,380 bp) and lower (50 bp) marker. Therefore, the concentration of *Lb. plantarum* 5BG in olives (CFU/g) or in brine (CFU/ml) was calculated on the basis of the number of identified colonies.

### Challenge Test

The challenge test was assessed to evaluate the effectiveness of *Lb. plantarum* 5BG to control and/or inhibit pathogens; a wild strain of *L. monocytogenes*, belonging to the culture collection of the Department of the Science of Agriculture, Food and Environment, University of Foggia, was used as the test organism.

*L. monocytogenes* was stored at -20°C in Nutrient broth (Oxoid), supplemented with 33% sterile glycerol (J.T. Baker, Milan). Before each assay the strain was grown twice in Nutrient broth incubated at 37°C for 24 h to attain an early stationary phase (ca. 8 Log CFU/ml). Then, the microbial culture was centrifuged twice at 8,000 rpm for 15 min at 4°C; the broth was discarded, and the pathogen suspended in a sterile saline solution (0.9% NaCl)

Olive bag samples of both SET1 and SET2 were inoculated with *L. monocytogenes* through a sterile syringe; the ratio inoculum/brine was ca. 1:10, in order to achieve an initial concentration of *L. monocytogenes* of 5 Log CFU/ml). Olive bags were stored at 4°C for 3 months. Both olives and brines were periodically analyzed to monitor the total mesophilic count, LAB, yeasts, as reported above, and *L. monocytogenes* on Listeria Selective Agar Base (Oxoid), supplemented with Listeria selective supplement (37°C for 24–48 h) (Oxoid). The analyses were performed in duplicate (two bags) over two independent experiments and repeated twice for each bag.

### Sensory Analyses

Table olives were subjected to a time intensity descriptive sensory evaluation according to the information provided ISO 13300-1 (2006), modified as follows. The panelists were selected based on their sensory skills (ability to accurately determine and communicate the sensory attributes of a food product) (Meilgaard et al., 1999). Prior to testing, panelists were trained into the sensory vocabulary and into the identification of attributes, by using samples of commercial olives. The panel members were asked to base their judgment evaluating color (spots, uniformity, intense and typical of the fruit), taste (intense and typical of the fruit, salty, acid and bitter), odor (typical of the fruit, acid-off odor, putrid, butyric, metallic and off odors), and texture attributes (hardness, crunchiness, and fibrousness).

The panel members were asked to give a score from 1 to 9 to color, texture, and odor; a score of five was set as the break point. The assignment of the score was based on the following hits:

1. Color: 9 = green color typical of the cultivar, 1 = dark brown
2. Texture: 9 = firm and consistent, 1 = extremely soft
3. Odor: 9 = characteristic olive odor, 1 = extremely off odor.

The panel members were also asked to give a score for the overall quality (where 9 = excellent, 8 = very good, 7 = good, 6 = reasonable, 5 = not good, 4 = disliked, 3 = bad, 2 = very bad, 1 = reject).

### Statistical Analysis

All data are presented as mean values ± standard deviation (SD). Microbiological data, expressed as Log CFU/g of pitted olives or Log CFU/ml of brines, and physico-chemical data were compared by using the one-way factor analysis of variance (ANOVA). Significant differences (*p* < 0.05) among groups were determined by using the *post hoc* LSD Fisher test. Paired comparisons were analyzed by Student’s *t*-test (*p* < 0.05).

The *L. monocytogenes* count was modeled through the equation of Weibull, as re-parameterized by Mafart et al. (2002):

$$LogN={LogN}_{0}-{\left(t/\delta \right)}^{p}$$

where *LogN* is the count over the time *t* (Log CFU/ml); *LogN*_0_ the inoculum (Log CFU/ml); δ, the first reduction time (day), i.e., the time for a 1 Log CFU/ml decrease of the bacterial population; p, the shape parameter (*p* > 1 downward curve; *p* < 1, upward curve).

The results were also fitted through the Weibull equation, modified by Bevilacqua et al. (2008) for the evaluation of the survival time:

$$\frac{LogN}{{LogN}_{0}}=1-{\left(t/s.t.\right)}^{p}$$

where s.t. is the survival time (days), i.e., the time after which the population is below the detection limit.

All statistical analyses were carried out using STATISTICA 6.0 software (StatSoft software package, Tulsa, OK, United States).

## Results

### Physico-Chemical Quality

**FIGURE 1:**
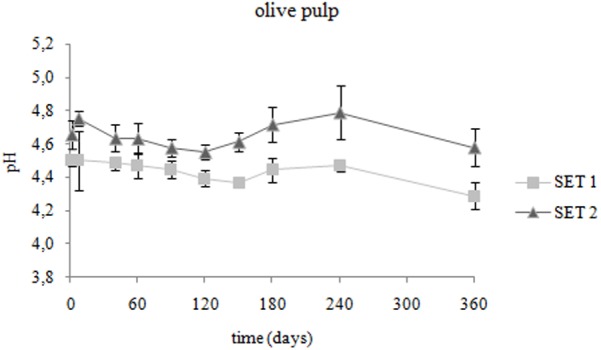
Changes in pH of Bella di Cerignola Spanish-style olives packaged in PTE bags (olive/brine ratio, 8/1): SET1 (bio-preserved), SET2 (control). Means and standard deviations were obtained from measurements made in duplicate packages from 2 different experiments (*n* = 4).

Spanish-style fermented Bella di Cerignola desalted olives provided by the industry were used in the present study (pH: 4.67 ± 0.07; firmness: 62.4 ± 11.6 N/g; color parameters L^∗^ 55.73 ± 3.40, a^∗^ -0.86 ± 0.34 b^∗^ 39.34 ± 4.13). The evolution of pH on olive fruits packaged in PE bags in brine during storage at 4°C is presented in **Figure 1**. At the onset of storage (T0), after the 5-days brining in the presence of the preserving strain, the olive pH lowered to 4.50 ± 0.03 (*p* < 0.05). In chemically acidified control SET2, the olive pH remained unvaried (*p* > 0.05). The pH of brines showed values ranging from 4.28 ± 0.02 to 4.27 ± 0.13 in SET1 and from 4.39 ± 0.03 to 4.55 ± 0.06 in SET2, respectively, at T0 and T360. During the entire storage period pH values of inoculated olives and brines were significantly lower (*p* < 0.05) with respect to chemically acidified control.

**FIGURE 2:**
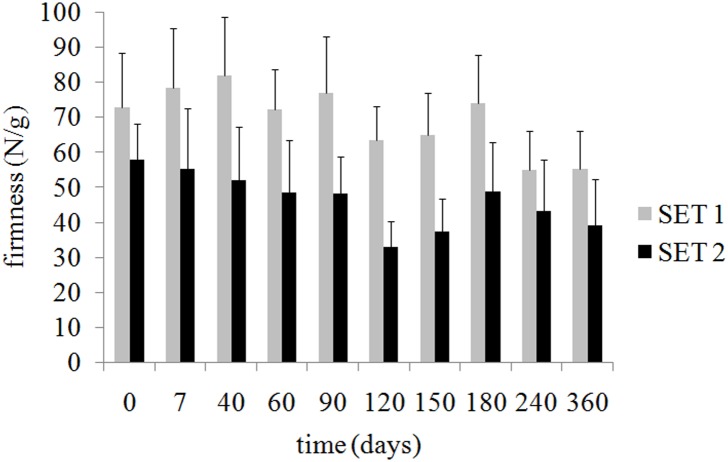
Instrumental firmness of bio-preserved (SET1, gray bars) and control (SET2, black bars) Bella di Cerignola Spanish-style olives packaged in PTE bags (olive/brine ratio, 8/1). Means and standard deviations were obtained from measurements made on three different olives analyzed from duplicate packages per each experiment (*n* = 12).

Instrumental firmness of olives is shown in **Figure 2**. At the onset of storage (T0), olive firmness remained stable (*p* > 0.05) in both sets. During storage, firmness of inoculated olives (SET1) remained stable (*p* > 0.05) until day 180, after that significantly decreased (*p* < 0.05). Whereas, in control SET2, firmness of olives significantly decreased already at day 120 (*p* < 0.05). At each sampling time, bio-preserved olives showed higher (*p* < 0.05) values of firmness compared to control olives.

**FIGURE 3:**
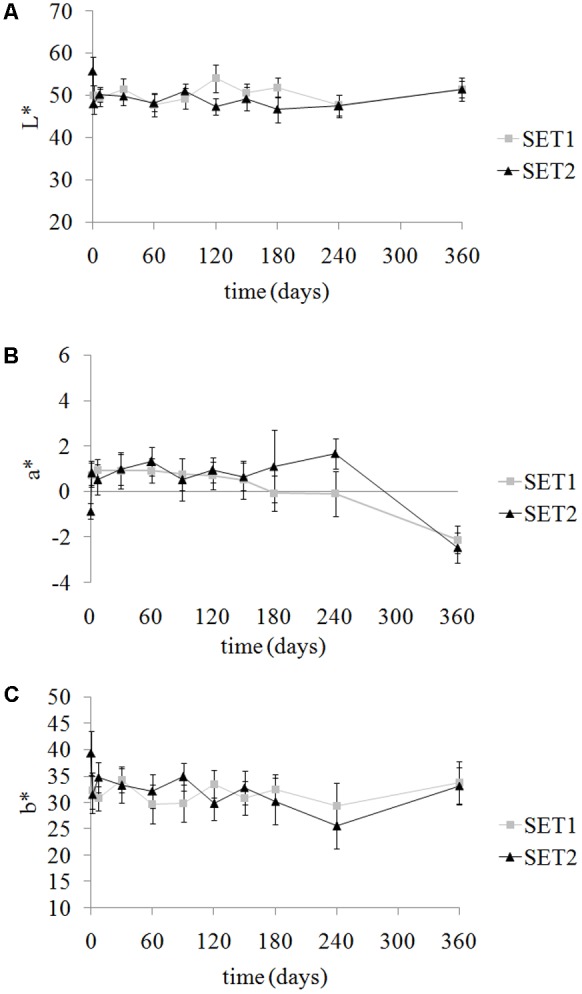
Evolution of color parameters Luminance L^∗^ **(A)**, green-red a^∗^ **(B)** and blue-yellow b^∗^ **(C)** in SET1 (bio-preserved), and SET2 (control) Bella di Cerignola Spanish-style olives packaged in PTE bags (olive/brine ratio, 8/1). Means and standard deviations were obtained from measurements made on nine different olives analyzed in duplicate packages per each experiment (*n* = 18).

Variation of color parameters of olives during storage determined comparable (*p* > 0.05) total color differences (ΔE^∗^) between sets with average values of 9.5 ± 2.4 and 10.3 ± 3.0 for SET1 and SET2, respectively. Although color parameters varied (*p* < 0.05) during storage, a similar trend was observed in both sets (**Figure 3**).

### Microbiological Quality

**FIGURE 4:**
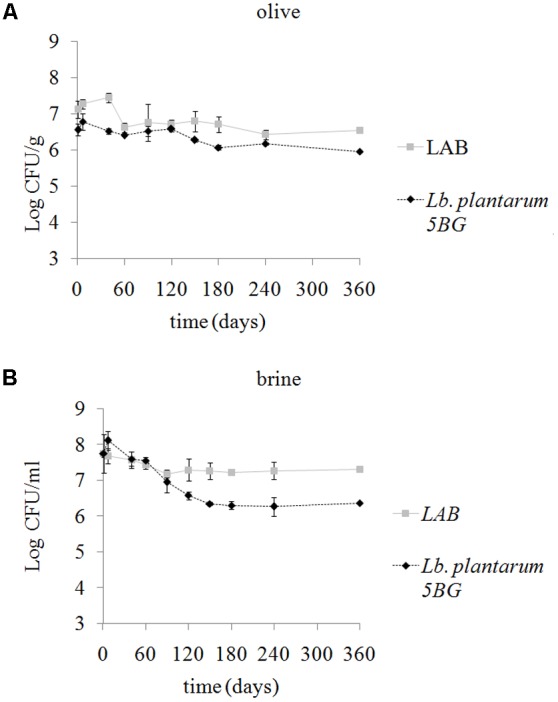
Changes of the LAB population and survival of the bio-preserving strain *Lb. plantarum* 5BG on olives **(A)** and in cover brines **(B)** of SET1 Bella di Cerignola Spanish-style olives packaged in PTE bags (olive/brine ratio, 8/1) and stored at 4°C for 1 year. Means and standard deviations were obtained from measurements made in duplicate packages from two different experiments (*n* = 4).

Significant differences in LAB populations (*p* < 0.05) were observed between SET1 and 2 having SET1 higher (*p* < 0.05) microbial loads during the whole experiments. In control SET2, LAB population ranged from 4.63 ± 0.73 to 6.05 ± 0.33 Log CFU/g in olives at T0 and T360, respectively. Values ranged from 5.04 ± 0.28 to 6.51 ± 0.11 Log CFU/ml in brines (data not shown). The presence of the *Lb. plantarum* strain resulted in an increase in total LAB in inoculated SET1 and populations remained stable throughout the experiment (**Figures 4A,B**). The REP-PCR profile of 148 presumptive LAB isolates from olives and brines of SET1 and SET2, were compared to the specific pattern obtained from the pure culture of *Lb. plantarum* 5BG. Bacterial load of the inoculated strain at T0 was 6.56 ± 0.17 Log CFU/g on olives and 7.74 ± 0.06 Log CFU/ml in brines (**Figures 4A,B**). The bio-preserving strain population remained stable on olives until the end of storage while a decrease was observed in brines. In control SET2 none of the REP-PCR profiles of the analyzed colonies was comparable to the *Lb. plantarum* 5BG pattern (data not shown).

**FIGURE 5:**
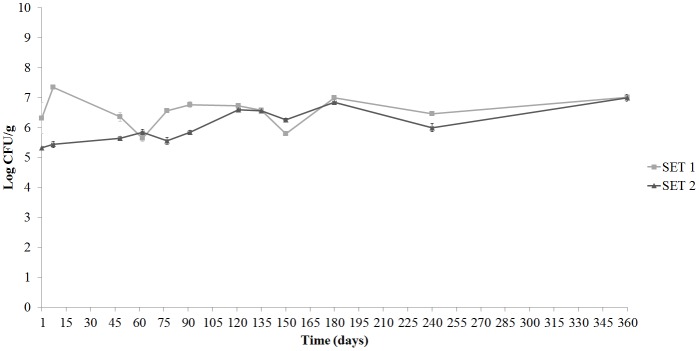
Evolution of mesophilic bacteria in Spanish-style olives pulp packaged in PTE bags and stored at 4°C: SET1 (bio-preserved), and SET2 (control). Means and standard deviations were obtained from measurements made in duplicate packages from two different experiments (*n* = 4).

Regarding the other microbial populations, the evolution of the total mesophilic count during olive storage is showed in **Figure 5**. A significant (*p* < 0.05) increase of counts was observed in control SET2, while counts remained almost unvaried (*p* > 0.05) in SET1 until 1 year. The corresponding values of the brines (data not shown) appeared to be higher than those found on the pulps during the entire observation period, reaching similar values, about 7.39 and 7.28 Log CFU/ml, respectively, for SET1 and SET2.

Along with the prevailing LAB populations monitored on olive fruits, yeasts were present in both sets during the whole experiment at average values of 5.25 ± 0.88 log CFU/g and 5.30 ± 0.42 log CFU/g, respectively, for SET1 and SET2 and never attained the break-point (6 log CFU/g). In brines a similar trend was observed with comparable (*p* > 0.05) yeast populations found in SET1 (6.34 ± 0.58 log CFU/ml) and SET2 (6.30 ± 0.67 log CFU/ml). Molds, staphylococci, and Enterobacteriaceae were always below the detection limit.

### Challenge Test

**FIGURE 6:**
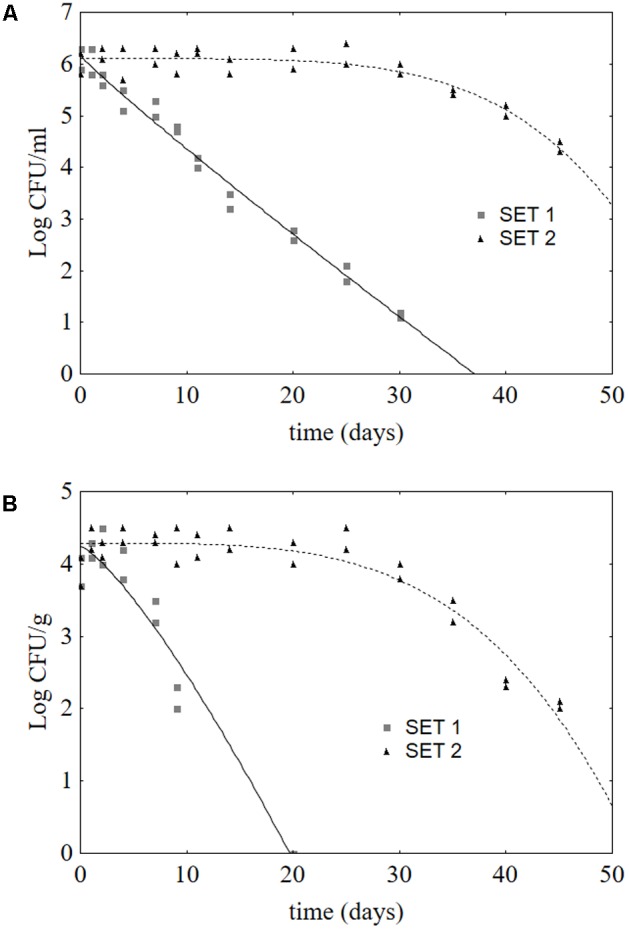
Death kinetics of *L. monocytogenes* in brines **(A)** and on olive surfaces **(B)**. The lines represent the best fitting through the Weibull equation: SET1 (bio-preserved) and SET2 (control).

The data of *L. monocytogenes* were fitted through the Weibull model to assess if the presence of *Lb. plantarum* could affect both the survival and the shape of the death kinetics (**Table 1** and **Figure 6**). **Table 1** reports the fitting parameters for SET1 and SET2 inoculated with the pathogen. The Weibull model is characterized by three main parameters, i.e., the initial pathogen count (LogN_0_), the first reduction time (δ) and the shape parameter (p). The first reduction time is similar to the *D*-value for the linear thermal death kinetic and can be used as a simple index of how a treatment affects the survival of the model microorganism. In brines, *L. monocytogenes* experienced a δ of 40.07 ± 0.91 days in the control SET2, thus suggesting a prolonged survival; however, the inoculation of *Lb. plantarum* created conditions that strongly affected *L. monocytogenes* and reduced δ to 5.30 ± 0.86 days. This reduced survival was the result of a significant change in the shape of the death kinetic, as suggested by the shift in the shape parameter (p). In SET2-brine, the shape parameter was 4.72 ± 0.79, whereas in SET1 this parameter was reduced to 0.94 ± 0.08 and the shape of the death kinetic completely changed.

**Table 1:** Weibull parameters (±standard error) for the death kinetic of *L. monocytogenes* in brines and on olive surface.

	LogN_0_	δ	*p*	*R*	*s.t.*
**Brines**					
SET1	6.17 ± 0.14	5.30 ± 0.86	0.94 ± 0.08	0.991	36.63 ± 1.29
SET2	6.10 ± 0.05	40.07 ± 0.91^∗^	4.72 ± 0.79^∗^	0.933	58.77 ± 3.70^∗^
**Olives**					
SET1	4.25 ± 0.19	6.37 ± 1.24	1.28 ± 0.20	0.972	18.43 ± 1.12
SET2	4.29 ± 0.06	35.70 ± 1.10^∗^	3.84 ± 0.49^∗^	0.950	50.90 ± 1.47^∗^


**SET1, inoculated with Lb. plantarum; SET2, control. LogN_0_, initial count (Log CFU/ml); δ, first reduction time (time to attain a 1-log decrease, day); p, shape parameter; s.t., survival time (day). ^∗^Significant difference between SET1 and SET2 (LSD test, P < 0.05).**

A modified approach of the Weibull equation, proposed by Bevilacqua et al. (2008), contains the survival time of the model organism. *L. monocytogenes* experienced a survival time of 58.77 ± 3.70 days in the control SET2 and 36.63 ± 1.29 days in the brine inoculated with *Lb. plantarum*. The same approach was used to model *L. monocytogenes* on olive surfaces. In the presence of *Lb. plantarum*, a significant reduction of δ (from 35.70 ± 1.10 to 6.37 ± 1.24 days), an effect of the shape parameter and a decrease of the survival time (from 50.90 ± 1.47 to 18.43 ± 1.12 days) were observed.

### Sensory Quality

**FIGURE 7:**
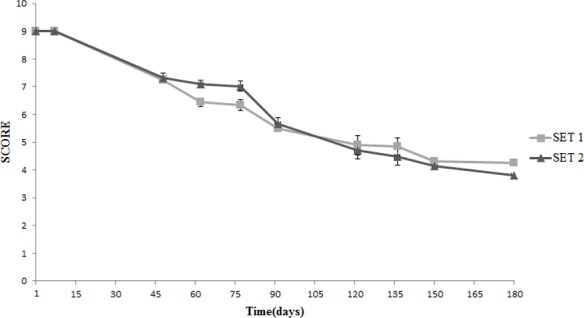
Sensory analysis: evolution of the color in Spanish-style olives packaged in PTE bags and stored at 4°C: SET1 (bio-preserved), and SET2 (control). Means and standard deviations were obtained from measurements made in duplicate packages from two different experiments (*n* = 4).

**FIGURE 8:**
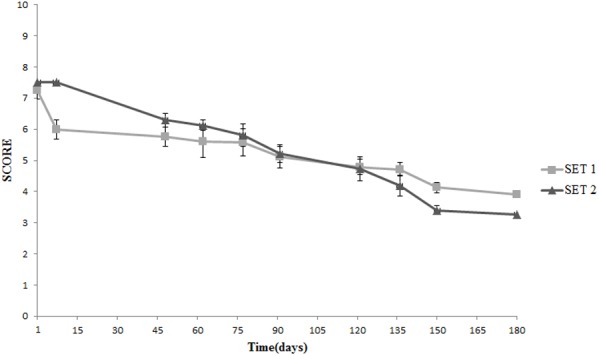
Sensory analysis: evolution of the odor in Spanish-style olives packaged in PTE bags and stored at 4°C: SET1 (bio-preserved), and SET2 (control). Means and standard deviations were obtained from measurements made in duplicate packages from two different experiments (*n* = 4).

Both SET1 and SET2 were positively accepted for color, having a uniform appearance and the characteristic color of Spanish-style olives, with slight differences between SET1 and SET2 (**Figure 7**). At the beginning of the storage, the score for odor was lower in SET1 as consequence of the supplementation of *Lb. plantarum*, but for this sample the panelists always recorded an equilibrated odor. On the other hand, they perceived alcoholic odors in SET2 since the 4th month onward and assigned a lower score (**Figure 8**). In SET1, panelists never found smells of butyric, acidic, rancid, and metallic like odors during the entire observation period.

**FIGURE 9:**
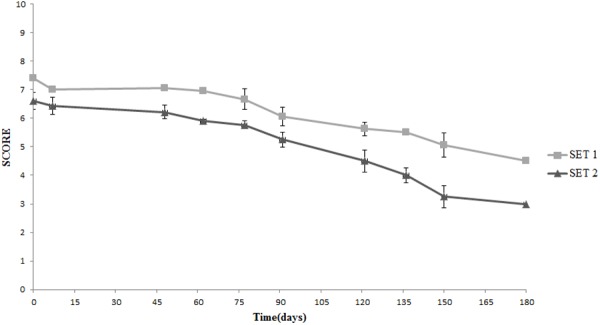
Sensory analysis: evolution of the texture in Spanish-style olives packaged in PTE bags and stored at 4°C: SET1 (bio-preserved), and SET2 (control). Means and standard deviations were obtained from measurements made in duplicate packages from two different experiments (*n* = 4).

**FIGURE 10:**
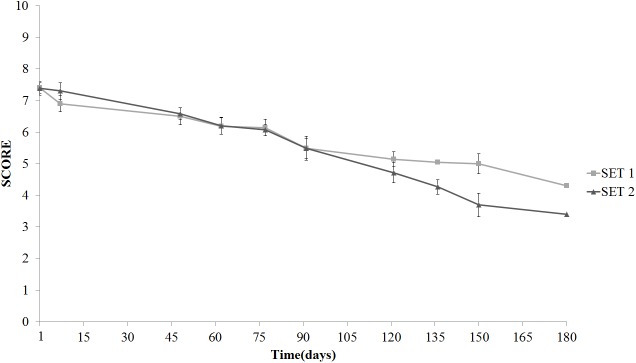
Evolution of the overall sensory quality in Spanish-style olives packaged in PTE bags and stored at 4°C: SET1 (bio-preserved), and SET2 (control). Means and standard deviations were obtained from measurements made in duplicate packages from two different experiments (*n* = 4).

Texture was the real discriminative parameter among samples (**Figure 9**). SET1 recorded, since the beginning and during the whole storage period, a firmer texture, that is a score significantly higher than in SET2, in line with the instrumental data shown in **Figure 2**. Furthermore, bio-preserved olives proved to be better also regarding crunchiness and fibrousness; in fact, panelists underlined a positive mouth texture in relation to the force required to crunch samples with the back molars. On the contrary, SET2 olives revealed a gradually softening with the visual detachment of the pulp. Bio-preserved olives appeared to be positively accepted also in relation to their taste, since they were perceived by panelists as more equilibrated with better scores in relation to acidity and bitterness. SET2 olives had a less intense taste with an excessive bitter and vinegary taste perceived by panelists. In terms of overall quality, reported in **Figure 10**, both samples revealed a gradual decrease; however, the control SET2 was rejected by panelists after 115 days while the bio-preserved olives recorded a longer storage (at least 5 months), thus reflecting the texture trends.

## Discussion

Packaging in plastic bags is a procedure currently adopted to store Bella di Cerignola Spanish-style olives using 8–10% brine (olive/brine ratio 4/1) in the presence of chemical acidifiers and antioxidants to ensure microbiological stability and sensory quality. However, long-term storage in these conditions cannot be achieved since sensory parameters, particularly texture, experience a strong decrease. In addition, the increasing consumer requirements for low levels of acidity and salt pave the way for the use of bio-protective cultures for a sustainable long-term preservation of table olives. It is noteworthy that the bio-preserving strain used in this study was selected among several strains belonging to *Lb. plantarum*, *Lb. pentosus*, *Lb. casei*, and *Lb. paracasei* spp., isolated from olive environment (Lavermicocca et al., 1998; De Bellis et al., 2010).

In fact, the bio-preserving strain, well adapted in the brine environment, replaced the indigenous LAB population, survived for 1 year and allowed to reach pH values lower than 4.3 in brine as required by the Codex Alimentarius standard (CODEX/COI, 2013).

The combined effects of microbiological and physico-chemical changes occurring during storage (pH, temperature, strain survival) are essential for olive preservation and the application of LAB at the onset of fermentation (Argyri et al., 2015; Blana et al., 2016) or at the moment of packing (Rodríguez-Gómez et al., 2014) represents a valid procedure to preserve Spanish-style table olive quality. Temperature influences the survival rates of LAB in brines and olives as reported in studies on Spanish-style olives, previously fermented with a *Lb. plantarum* strain, thermally treated and packaged in the presence or not of brine (Argyri et al., 2015; Blana et al., 2016). In these studies, during storage at 4°C, a high survival rate of a *Lb. plantarum* strain (96 and 94.1% recovery after 6 months) and no survival or a very low recovery (13.3% after 1 year) has been observed on olives packaged in brine or modified atmosphere, respectively. In our study the bio-preserving strain was able to survive at 4°C under packaging conditions for a long period (1 year) in brines and on olive surface replacing the indigenous LAB population without the need of thermal treatments that can influence the sensory profile. Moreover, the presence of LAB populations can improve the microbiological quality of table olives (Rodríguez-Gómez et al., 2013; Bonatsou et al., 2017). In our study, the total absence of *Enterobacteriaceae*, Staphylococci and molds could be related to the successful long term persistence of the *Lb. plantarum* strain (SET1) that might have hampered the growth of alterative microorganisms and to the chemical acidification in control SET2. The *Lb. plantarum* strain can contribute in preventing spoiling, hence reducing the need for chemical preservatives or thermal treatments in low-salt products. During storage, yeasts were present in brines and on olive surface at high loads in both sets. These microorganisms are known to contribute to the flavor of table olives, however, in some conditions they may be considered spoilage microorganisms (Arroyo-López et al., 2008).

In order to assess if the microbiological and physico-chemical conditions obtained in the applied bio-preserving procedure, could hamper the growth of microbial pathogens, *L. monocytogenes* was chosen as a model since previous researches indicated the prolonged survival of this pathogen on table olives and brine (Bevilacqua et al., 2018). Generally, acid-adapted cells of *L. monocytogenes* can survive during shelf life in acidified or fermented products (Caggia et al., 2004, 2009; Tassou et al., 2009). As a result, in our study the pathogen load remained unvaried on control olives (SET2) until about 30 days. In *Lb. plantarum* bio-preserved olives, a dual effect on *L. monocytogenes* was observed: the decrease of the first reduction time and the change in the shape from a downward to a linear kinetic. These two effects were probably related to an increased death rate resulting from the production of antimicrobial compounds and the antagonistic ability of the strain, as well as to the reduced pH in SET1. Some preliminary experiments for the strain of *L. monocytogenes* used in this paper, in fact, revealed that it could survive a pH 4.0–4.5; however, the combination of acidic pH, low-salt amount and refrigeration could enhance and contribute to the antimicrobial effect of *Lb. plantarum* 5BG. As previously reported, *Lb. plantarum* 5BG, antagonized the human pathogen *Yersinia enterocolitica* in coculture (Lavermicocca et al., 2008) and was able to produce antimicrobial compounds and in particular organic acids responsible for antifungal properties (Valerio et al., 2016). Moreover, the technological suitability of *Lb. plantarum* 5BG was already demonstrated in green and black olive fermentation processes (Lavermicocca et al., 2002).

The evaluation of sensory quality was essential for the final determination of olive shelf life since no microbial alteration was found in any of the experimental samples. A brilliant green color is of great importance for Bella di Cerignola olives. In industrial practice the addition of ascorbic acid is common to prevent oxidation (López et al., 2005; Segovia-Bravo et al., 2011) and it has been demonstrated that it positively affects fruit color regardless the presence of preservatives (Casado et al., 2010).

The correlation between pH values and olive color is well known since low pH values are associated with a light color (Garrido-Fernández et al., 1997). Thus, the lower pH of SET1 could be related to good score for color, as also confirmed by the instrumental measurements. Bio-preserved olives showed changes similar to control olives preserved in chemically acidified brine containing ascorbic acid.

Finally, the taste was the other relevant sensory parameter better preserved in bio-preserved olives. Although the score in the initial part of the storage was lower in SET1 samples, the panelist always recorded an equilibrated taste without any excessive bitter or acidic perception probably due to the β-Glucosidase activity of *Lb. plantarum* 5BG, which is able to hydrolyze bitter glucosides into no bitter compounds (Lavermicocca et al., 1998). On the contrary, in SET2 samples, above all at the end of storage, the panelists underlined a vinegary and ethanolic perception, responsible for the lower score after 115 days.

## Conclusion

This paper represents a valuable contribution for a possible scale-up of bio-preservation of table olives, in order to avoid chemical preservatives and/or detrimental thermal treatments. The approach hereby proposed is based on the use of a bio-preserving *Lb. plantarum* strain able to replace wild microbiota and persist at concentration higher than 6 Log CFU/g on packaged Spanish-style olives during refrigerated storage. The environmental stress conditions generated in olives – low pH values, low storage temperature, presence of the bio-preserving strain – influenced the death kinetic of *L. monocytogenes.*

Results obtained in this study are promising even if further efforts are required to validate this approach on other olive varieties.

## Author Contributions

PL, AC, MDN, and MC conceived of the study. PL, AC, AB, and MC designed the experiments. LA, SL, FV, MP, and AB performed the experiments. PL, LA, FV, AB, MC, and AC interpretated the results. PL, MC, and AC funded the research. All authors wrote and approved the manuscript.

## Conflict of Interest Statement

The authors declare that the research was conducted in the absence of any commercial or financial relationships that could be construed as a potential conflict of interest.
